# Book Notices

**Published:** 1892-12

**Authors:** 


### Book Notices.

PHYSICIANS' VISITING LIST, 1893. Lindsay & Blakiston's. The JOURNAL is in receipt of this old stand-by, which, for forty years, has held prominent place in the ranks of such publications. It is about as near perfection as a pocket record book for physician's use will ever be brought. We cannot see wherein it could be improved. It is much in little, truly, and besides being a record book, it contains tables and data for reference in almost any emergency. Price, $1.00.

The JOURNAL acknowledges the courtesy of a copy of the Transactions Texas State Medical Association for 1892, in extra morocco binding and gilt, with the editor's name in gilt letters. The JOURNAL makes its bow to Secretary West, and extends sincere congratulations on the excellent manner in which the volume for 1892 has been issued. It is truly creditable, alike to the publishing committee, and the State Association. Of its contents, we will speak later.

A MANUAL OF THE PRACTICE OF MEDICINE, prepared especially for students. By A. A. Stevens, A. M., M. D., Instructor of Physical Diagnosis University Pennsylvania, etc. Illustrated. W. B. Saunders, Publisher, Philadelphia. Cloth, 500 pages, Price $2.50.

This is an outline of general practice written at the request of students of the University of Pennsylvania, and intended for students preparing for examination. There is much useful knowledge in a small space and the book will serve, doubtless, the purpose for which it was written.

THE JOURNAL is in receipt of Gould's Pocket Dictionary, containing 12,000 medical words, pronounced and defined. Published by P. Blakiston & Co., Phila. Morocco cover. Price $1. A very valuable pocket companion—useful to all doctors.

MEDICAL REVIEW VISITING LIST for 1893. This old standby is the first to make its appearance. The price is only 75 cts. Nearly all doctors are familiar with it and appreciate its many conveniences.

PEPPER'S new book "A Treatise on the Theory and Practice of Medicine, by American Teachers," will be out in January, i.

e., vol. 1 will be; and vol. 2 will appear a few weeks later, so the JOURNAL is informed by the enterprising publisher, W. B. Saunders, Philadelphia. Will be sold by subscription only. Cloth, $5; sheep, $6. Everybody will want Pepper's new book.

---

DISEASES OF THE LUNGS, HEART, AND KIDNEYS.—By N. S. Davis, Jr., A. M., M. D., Professor of Principles and Practice of Medicine, Chicago Medical College; Physician to Mercy Hospital; Member of the American Medical Association, Illinois State Medical Society, Chicago Medical Society, Chicago Academy of Sciences, Illinois State Microscopical Society; Fellow of the American Academy of Medicine; Author of "Consumption, How to Prevent It and How to Live With It," etc. No. 14 in the Physicians' and Students' Ready-Reference Series. In one neat 12mo volume of 359 pages. Extra cloth, $1.25 net. Philadelphia. The F. A. Davis Co., 1231 Filbert Street.

This book has been most favorably received by the profession, and the medical press are almost unanimous in commending it.

---

DISEASES OF THE EYE, a Handbook af Ophthalmic Practice, by G. E. de Schweinitz, M. D., Professor of Diseases of the Eye, Philadelphia Polyclinic; Ophthalmic Surgeon to Children's Hospital and to the Philadelphia Hospital; Ophthalmologist to the Orthopædic Hospital and Infirmary for Nervous Diseases; Lecturer on Medical Ophthalmoscopy, University of Pennsylvania, etc. Forming a handsome royal 8vo. volume of more than 600 pages. Over 200 fine wood cuts, many of which are original, and two chromo-lithographic plates. Price, cloth, $4; sheep, $5. W. B. Saunders, Publishers, Philadelphia, Pa.

The object of this manual is to present to the student and practitioner, who is beginning work in the field of ophthalmology, a plain description of the optical defects and diseases of the eye. To this end special attention has been paid to the clinical side of the question; and the methods of examination, the symptomatology leading to a diagnosis, and the treatment of the various ocular defects have been brought into special prominence. Anatomy, physiology, and pathological histology, in so far as they serve the purpose just stated, have been omitted. The sections devoted to optical principles and the normal and abnormal refraction of the eye in large portion, have been written by Dr. James Wallace, Chief of the Eye Dispensary of the University Hospital. The chapter devoted to the application of the shadow-test has been prepared by Dr. Edward Jackson.

